# Controlling Single-Emitter Strong Coupling by Sculpting DNA Dye Scaffolds in NPoM Cavities

**DOI:** 10.1021/acs.jpcc.5c00278

**Published:** 2025-02-05

**Authors:** Sara Rocchetti, Thieme Schmidt, Ulrich F. Keyser, Jeremy J. Baumberg

**Affiliations:** † Nanophotonics Centre, Cavendish Laboratory, University of Cambridge, Cambridge CB3 0HE, U.K.; ‡ Maxwell Centre, Cavendish Laboratory, University of Cambridge, Cambridge CB3 0HE, U.K.

## Abstract

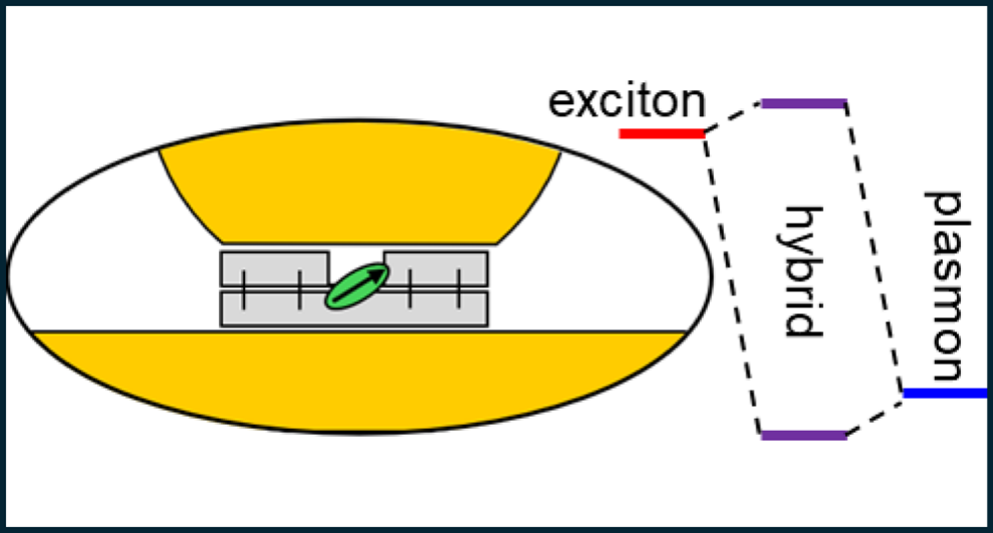

Coherent coupling of light and single molecules enables the development of next-generation room temperature-capable nanophotonic devices. Small mode-volume optical fields can be achieved with plasmonics, but challenges remain in placing oriented emitter molecules inside plasmonic cavities to access strong coupling consistently in emission. Using DNA origami, single-emitter molecules can be aligned inside subnanometric cavities created between a gold nanoparticle and a gold mirror. We observe that the exact design of DNA scaffolding architecture surrounding a cyanine dye changes how its emission couples to the nanocavity, as well as how Au atoms respond to the optical forces, leading to continuous tuning of the dominant plasmonic mode. Through this, we show how strong coupling between three different dyes and the plasmon resonance always leads to low-energy light emission, independent of detuning.

## Introduction

In recent years, the study of plasmon-exciton interactions at the single-molecule level has gained significant interest because of potential applications in enhancing optoelectronic devices, improving sensing technologies, and accessing quantum technologies at room temperature.^1^ Creating strongly coupled mixed states between individual emitters and visible light poses a significant challenge due to the 100-fold difference in their spatial scales. This size mismatch can be avoided by confining light inside deep subwavelength-sized nanocavities through exploiting metal plasmons, thereby achieving strong coupling.^2^

**Figure 1:**
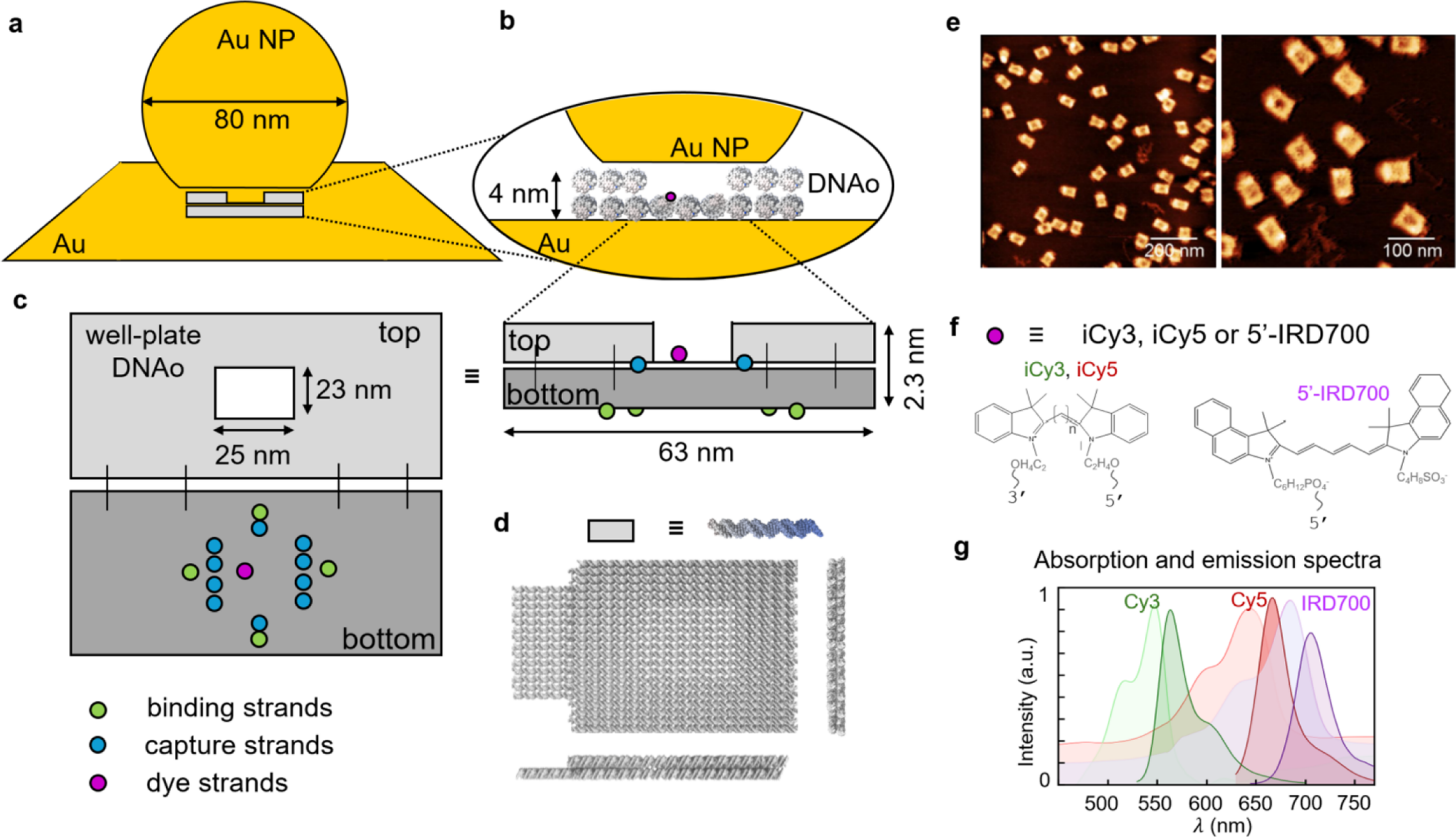
Well-plate DNAo structure in nanoparticle-on-mirror (NPoM). (a) Schematic of well-plate DNAo scaffolding the nanogap of a NPoM cavity (not to scale). The inset in (b) shows the location of one single-emitter site (purple) on DNAo in the nanogap. (c) Bottom layer (dark-gray) of DNAo structure functionalized with binding strands (green) to allow immobilization onto gold mirror, capture strands (blue) to fix AuNP onto the origami, and emitter binding strands (purple). (d) 3D rendering of the final structure. (e) AFM images confirm the integrity of the DNA constructs on mica. (f) Molecular structures of internal cyanine dyes (iCy3, iCy5) and singly attached IRD700 dye (5′-IRD700). (g) Solution absorption (light shading) and fluorescence spectra (dark shading) of each dye.

Accessing the single-molecule emission regime for strongly coupled systems requires a substantial reduction in the typical number of molecules involved and combining robust precision placement with ultrasmall plasmonic optical volumes.^3^ While self-assembled nanocavities can now routinely reduce optical mode volumes^4^ (which parametrize the effective cavity round trip time for strong-coupling) below 100 nm^3^,^3,5^ further strategies to couple with a single ∼ nm^3^ emitter are required to create systems that do not also damage under irradiation. To address this challenge, we use DNA nanotechnology in conjunction with a nanoparticle-on-mirror (NPoM, Figure 1a,b) geometry to explore how different dye molecules behave in such strong-coupling nanocavities. Recent advances in single-molecule confinement using DNA origami have highlighted promising results through split peaks observed in light scattering.^6−8^ However, to date, the photoluminescence spectra from single-emissive molecules in nanogaps have been somewhat inconsistent; hence, the present study. We also note related work using single semiconductor colloidal quantum dots in plasmonic nanocavities,^9−12^ which give rather different results compared to our single molecule emission, and highlight the possible differences from their much larger dipole size, isotropic dipole orientation, 3-fold degeneracy, and influence of a number of dark states.

We thus explore here how the precision and flexibility of DNA nanotechnology can avoid previous problems that led to gold facet destabilization by using an alternative well-plate DNA-origami (DNAo) design. This provides excellent spatial alignment, which is critical for observing strong coupling, although we note that orientational control of the emitter alignment is not yet solved. These well-plate scaffolded NPoM cavities are measured through dark-field (DF) scattering spectra that characterize the resulting architecture. The corresponding single-molecule photoluminescence (PL) spectra show strong coupling for a variety of different detuning regimes using molecules with different emission energies and reveal the influence on PL of mixing electronic states with the plasmon even for detunings >0.5 eV.

## Methods

### DNA Origami Folding and Purification

Single-stranded viral DNA scaffold (7249 nucleotides) isolated from the M13mp19 derivative is folded into rectangular DNA origami structures in 12 mM MgCl_2_ and 1× TE buffer and purified from excess staples using centrifugal filtration. Further details on the folding and all experimental conditions for creating these structures can be found in our previous work.^13^

### Gold Nanoparticle Functionalization and NPoM Assembly

AuNPs (D = 80 nm) are functionalized with an excess of single-stranded DNA (thymine, 14 nucleotides) carrying a dithiol group on their 5′ end side. The protocol is described in our previous work.^13^ Purified DNA nanostructures (2 nM) are immobilized on a template-stripped gold film via their thiolated strands. A hydrophobic layer of dodecanethiol in ethanol (1 mM V/V) is used to passivate the free Au surface. DNA-functionalized AuNPs are then drop-cast on the DNAo structures and left to hybridize for at least 10 min, before being rinsed away.

### Single Nanoparticle DF and SERS Measurements

Both DF and SERS spectra are recorded on a home-built confocal Raman microscope. The setup features and details are described in our previous work.^13^

## Results and Discussion

The well-plate DNA origami structure, designed with the software caDNAno, is composed of 42 helices arranged in a double layer (Figure 1c,d). Unlike previous rectangularly shaped constructs, which used a full double layer,^13^ the central portion of the top layer is now removed to provide a 23 × 25 nm^2^ aqueous well in proximity to the dye. For all details of the design and its assembly in solution and onto the Au, we refer the reader to our previous publication.^13^ The excised scaffold is shifted in this design into unbound double helices lying outside the perimeter of the construct (Figure 1d). The bottom layer of the DNAo is modified with thiolated strands (green in **c**), which bind the nanostructure onto a gold film via covalent bonds, and polyadenine strands (blue in **c**), which capture polythymine-coated AuNPs (capture strands). Emitter molecules (purple in **c**) are bound at both their ends onto the lower layer but facing upward, inside the central well. The 3D rendering of the final structure (Figure 1d) is well matched by AFM images (Figure 1e), confirming the correct assembly of the DNAo structure, with homogeneous and well-separated constructs on the mica surface.

Compared to previous studies using full plates of DNAo, emitter molecules are now exposed to an aqueous solution near the metal nanoparticle facet (Figure 1b), rather than being screened by Mg^2+^ ions bound to the phosphate backbone of DNA in between. This is found to enhance strong coupling interactions between the dye molecule (ω_*X*_) and the confined cavity mode (ω_*C*_). Here, we compare three dyes (Figure 1f) with different solution absorption/emission wavelengths (Figure 1g) to investigate the influence of detuning Δ = ω_*X*_*– ω*_*C*_. While the two cyanine dyes (Cy3 and Cy5) are linked as an internal modification on a single DNA strand, which binds into the DNAo on both ends, the IRD700 dye has a single attachment from the 5′-end of a single DNA strand.

In previous studies,^5,13^ we demonstrated the significant differences in emission properties of single dyes inside full-plate DNAo when in solution compared to when embedded in the NPoM geometry. However, a significant unexpected feature was the enhanced light-induced instability of the facets. This arises because electronically resonant molecules generate large optical forces capable of extracting gold atoms from the nearby metallic surfaces.^14^ This creation of chains of adatoms induces extremely strong new vibrational lines (“picocavities”), which dominate the emission spectra, arising from surface-enhanced resonant Raman signals (SERRS) from single dyes.^15^

**Figure 2:**
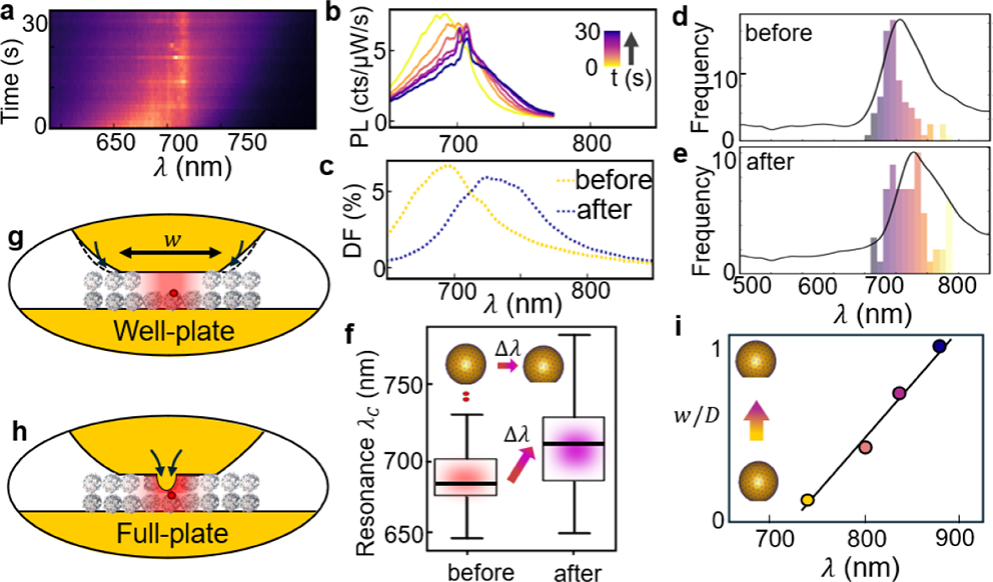
(a) Evolution of emission with time from a single NPoM cavity containing a Cy5-labeled well-plate DNAo construct, pumped by λ_p_ = 633 nm laser light for 30 s. (b) Selected emission spectra shows gradual redshift (yellow to purple = 0–30s) and increasingly intense Raman peaks at ∼690–710 nm. (c) Corresponding dark-field spectra before (yellow) and after (purple) laser illumination. (d,e) Histograms of DF resonant λ_*C*_ for >100 NPoMs before and after laser illumination, showing consistent redshift in the plasmon mode. Black curves are average DF spectra of the mean bin. (f) Whisker boxplot of λ_*C*_ shows 35 nm mean redshift (horizontal bars, arrow Δλ) after 30 s laser illumination. (g,h) Light-induced nanoparticle facet resculpting inferred for well-plate (g) and full-plate (h). (i) Simulated shifts from growth in facet size *w* (normalized by NP diameter *D*).

Here, by contrast, the well-plate DNAo nanogap spacer appears to produce much more gentle facet restructuring, comparable with previous experiments using robust MoS_2_ and molecular monolayer spacers.^16,17^ Emission (PL) spectra are collected using continuous illumination (λ_*P*_ = 633 nm, 150 μW·μm^–2^) for 30 s (Figure 2a). After background subtraction, the individual spectra show sharp Raman peaks together with a broad PL band, which redshifts over time (Figure 2b). While initially the PL maximum is close to the solution Cy5 emission at 670 nm, it saturates around 710 nm, enhancing the Raman peaks (which match those from the dye and the adenine in DNA^18^ when it crosses them), as expected from SERRS. To show these redshifts are controlled by the plasmon resonance, we record dark-field scattering spectra before (yellow) and after (purple) laser illumination (Figure 2c), which show a similar shift of the coupled mode Δλ_*C*_.

To quantify this evolution of scattering peak position, we perform automated measurements on a large number of particles (>200 NPoMs), with the histogram of spectral peak positions generally shifting after illumination (Figure 2d,e). Although both distributions are spread over σ*_c_* = ± 30 nm, their mean position shifts by Δλ_*C*_ ≈ +35 nm, as seen also in a whisker boxplot (Figure 2f) of the average shifts.

The light-induced redshifting of nanocavity plasmonic resonances has been observed in many experiments on such NPoM systems.^16,17^ Its origin has been identified as arising from the restructuring of the lowest facet on near-spherical nanoparticles, and it is strongly retarded (or disappears) when the facet cannot restructure in this way, for instance, using nanodecahedra with fixed (111) triangular facets^19^ or nanocubes with fixed (100) square facets.^16^ In neither case can the facets broaden in size due to the geometry of the nanoparticle, and indeed, the light-induced redshfting is now absent. This agrees with our previous work with DNA nanostructure-based NPoMs,^13^ which implied that these redshifts do not arise from the compression of the nanogaps as water is expelled from the DNAo, as previously suggested,^20^ because no difference is seen when our samples are immersed throughout in water. Similar shifts are also seen for completely incompressible 2D semiconductors in the nanogap.^17^

The cause of the redshift is thus a slow migration of atoms around the side of the nanoparticle (due to a combination of strong light-induced van der Waals attraction, which destabilizes surface atoms, and enhanced thermal diffusion). This increases the facet area (Figure 2g), lowering the resonant energy of the system. This facet widening is rather different from the previous restructuring found with DNAo full-plates, as inferred from the picocavity SERRS (Figure 2h), which suggested Au atoms were pulled right inside the DNAo structure.^13^ The more gentle facet growth here saturates in time due to the limiting wetting angle of the Au facet on the DNA set by the Au-DNA surface energy,^17^ as seen also for Au nanodimers with thiol molecular spacers.^21^ Nanocavity plasmon modes are sensitive to variations in the AuNP facet size,^16,22^ allowing extraction of the cavity geometry by observing the coupled mode scattering positions.^19,22^ We estimate the gap and facet size before and after laser illumination by comparing the scattering coupled mode peak position with previously published electrodynamics simulations,^22^ which also include details of these full electromagnetic calculations (that have been verified against experiments). These simulations imply an initial gap size of ∼4 nm, consistent with the double-layer DNA plate in the gap, and a facet diameter of *w* = (0.25 ± 0.05)*D*; for spherical nanoparticle, diameter *D* = 80 nm. After laser illumination, the gap is estimated to be ∼3 nm with a facet size of (0.55 ± 0.05)*D*, thus corresponding mainly to facet expansion. Simulated scattering spectra for nanoparticles with facets of increasingly truncated spheres (Figure 2i) reproduce these redshifts.^23^ We also note sporadic transient broad emission events (Figure 2a), known as “flares” that have recently been identified as optical delamination of the top monolayer of Au atoms, and that may contribute to the slow atomic migration.^24^

In summary, we find here that removing DNA helices from the immediate vicinity of the dye molecule changes the type of optically induced movement of Au atoms. When dsDNA surrounds the dye, the forces are sufficient to suck many Au atoms from the facet into its vicinity. On the other hand, when there is a pocket of water in the DNAo for the dye, these forces seem to be greatly reduced, and only the typical facet widening is seen. However, the cause of these differences is not yet clear. One possibility is that the polarizability of the solvated dye molecule can be reduced by ionic screening (even at optical frequencies), which in our models^14^ would reduce the induced forces on the Au atoms. When DNA shrouds the dye, this screening may not be possible (since the DNA binds the ions tightly). In the current situation, it shows the importance of the local environment of the dye (as in natural energy transfer cascades in rhodopsin, chlorophyll, and other biophotonic protein complexes^25^), which is accessed through DNAo design. This thus opens up a wealth of possibilities for further optimizing chromophore-metal interactions in DNAo-scaffolded constructs.

**Figure 3:**
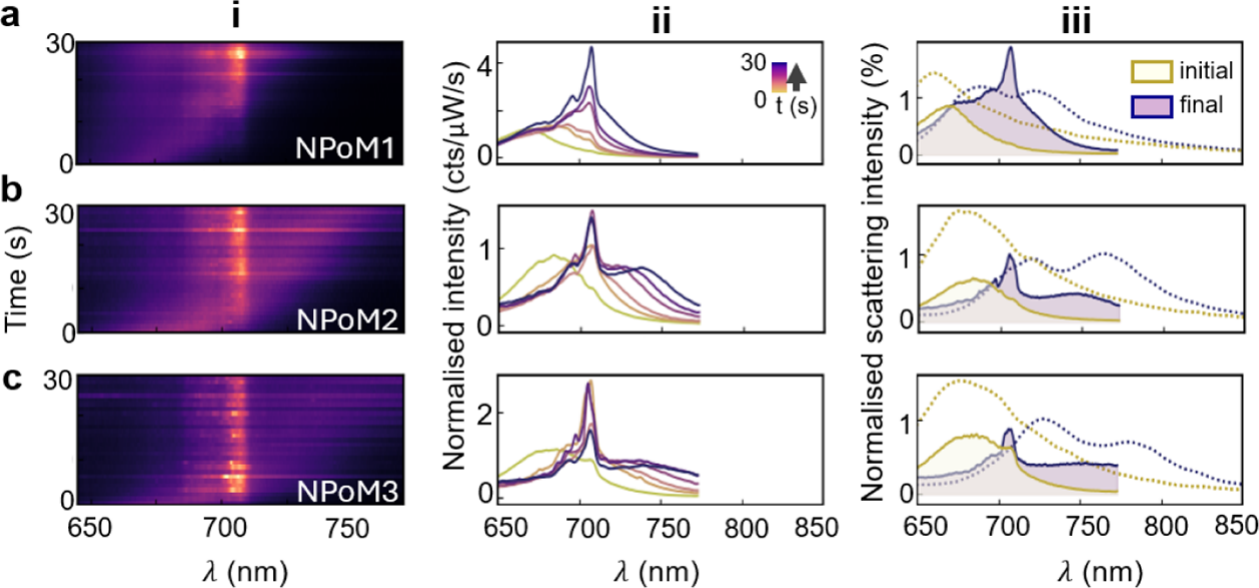
(a–c) Emission from three NPoM cavities containing a single Cy5 emitter on the well-plate DNAo. (i) Spectral maps, and (ii) individual spectra over 30 s of illumination. (iii) Initial (yellow) and final (purple) spectra from emission (filled solid) and dark-field spectra (dotted), showing splitting in scattering spectra after redshift of plasmon.

We now explore the spectra that arise from the emission of the dye molecule in this plasmonic environment. The slow tuning of the plasmon mode is extremely useful for examining the light-matter coupling since it allows (one-way) scanning of the detuning for each dye-plasmon construct (Figure 3). Intriguingly, as the plasmon redshifts from blue-detuning to resonance (Δ ∼ 0), a splitting in the scattering peak often appears (more than a third of the time). Differently from Figure 2, in these cases, an evident dark-field splitting is apparent by *t* = 30 s (purple), of magnitude Ω ∼ 110 meV (Figure 3iii dashed). Comparing the emission spectra (solid) also shows in this case a peak between the two split DF modes (partly masked by the strong SERRS peaks), also evident in Figure 3ii. At *t* = 0 s, however, this emission is slightly to the red side of the single DF peak observed (yellow curves), by on average 22 nm across many NPoMs, which likely arises from the spectral separation between near-field and far-field resonances.^26^

As shown in the simulations below (based on proposals in ref. (12, 27) , these features are expected for the strong coupling regime when the line width Γ is comparable to the Rabi splitting Ω because of the way that the DF and PL of the split polaritons interfere oppositely in the far field. Illuminating through the cavity mode (for DF) produces destructive interference (hence partially canceling to form a dip between ω_±_), while exciting nonresonantly via the exciton (for PL) gives constructive interference (obscuring the polariton splitting for Γ ∼ Ω at Δ = 0). The single-molecule Rabi splitting of Cy5 (∼100 meV with μ = 0.9D)^28^ observed here is 3-fold smaller than that for methylene blue molecules (∼300 meV, μ = 3.8D),^5^ as expected from their relative transition dipole moments μ. However, in the current realization, precision of the number of dye molecules is ensured, making this a robust quantum-emitting construct at room temperature. Besides gentle restructuring of the plasmonic facet (which saturates after ∼30s), the dye emission does not bleach, as previously noted, because Purcell-enhanced re-emission is so fast in this NPoM that the molecule is vanishingly often found in the excited state.^29^ The differences between these observations and previous experiments with larger gaps^6−8^ (which show conventional quenching effects but do not evidence the spectral changes seen here) emphasize the effect of tighter confinement on the light-matter interactions.

For near-zero detuning using Cy5 in these constructs, the Rabi coupling has to exceed the absorption line width and plasmon cavity line width for strong coupling.^30,31^ In the intermediate regime here, splitting can be seen in DF but not in PL; however, the real-time cavity retuning allows this to be verified for each construct. To further explore the strong coupling regime, we use the versatility of DNA nanotechnology to place differently detuned emitters inside the NPoM cavity, using Cy3 and IRD700 with emission wavelengths of 532 and 702 nm (Figure 1f,g).

**Figure 4:**
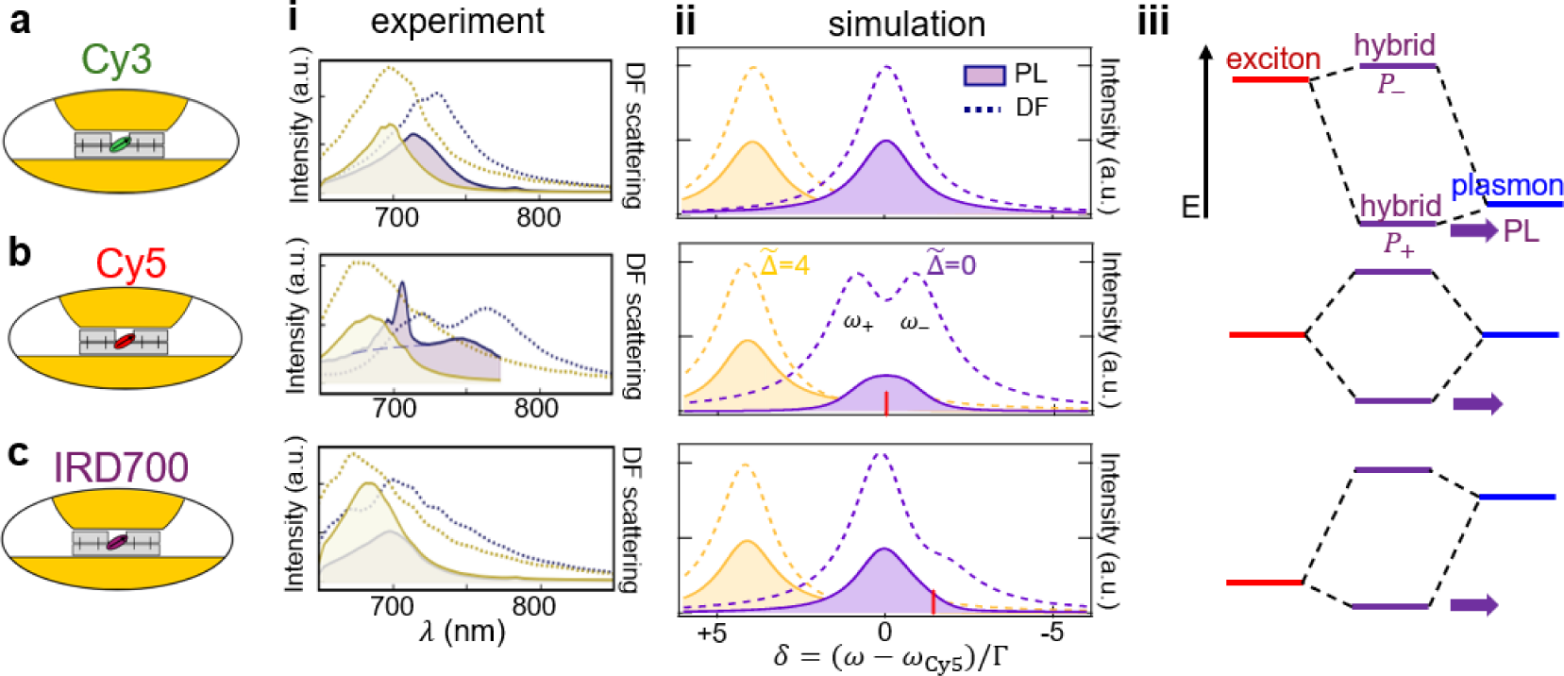
Comparing DNA origami NPoMs incorporating a single (a) Cy3, (b) Cy5, or (c) IRD700 molecule. (i) Emission (solid) and dark-field (purple) spectra at *t* = 0 s (yellow) and *t* = 30 s (purple). Dashed line in (b,i) shows PL without SERRS peaks of ∼710 nm. (ii) Simulations of dark-field and emission spectra at different normalized plasmon detunings , relative to the Cy5 transition (ω_CY5_). Transition dipoles 0.9, 0.9, and 0.4D for (a–c). (iii) Energy-level diagram showing exciton and plasmon energies for each emitter-plasmon pair.

Despite their very different emission wavelengths, surprisingly strong emission is always observed in the vicinity of the resonant plasmon for these well-plated NPoM cavities (Figure 4). For each molecule (a–c), we show exemplary spectra both initially (yellow) and after the facet growth has redshifted the plasmon (purple) for both PL emission (solid) and dark-field (dotted) spectra (Figure 4i). Only for Cy5 is the strong coupling directly seen in DF after the plasmon tunes into resonance.

To tentatively understand why nonresonant molecules emit at the plasmon wavelength, we consider the strongly coupled plexciton (hybrid plasmon-exciton) for the parameters of our experiment. Using the standard strong-coupling Hopfield model^32^ gives simulated spectra that match the experimental data reasonably well (Figure 4ii). In this picture, the reason why, despite its larger detuning, the Cy3 still emits strongly is due to the hybridization of the exciton with the plasmon (Figure 4iii). Despite a small energy shift in the lower plexciton *P*_+_, the mixing in of the exciton component allows efficient emission of this quasiparticle (purple arrows). In all cases, emission to the lower polariton (plexciton) state is rapid, so that it dominates absorption and emission.

Only for the resonant detuning case with Cy5 is intense SERRS observed, which is maximized for  = 0. In this case, the branching ratio between PL and SERRS becomes near unity, showing that relaxation within the excited electronic vibrational manifold can be slower than photon emission via plexcitons to the ground-state vibrational manifold. In all other cases, Kasha’s rule is observed, and nonresonant SERS is too weak to be seen. It is also apparent that the plasmon shifts are stronger for the resonant Cy5 molecule than the weaker-coupled Cy3 and IRD700, confirming that resculpting of the Au facet depends on the resonant polarizability of the single molecule inside the nanogap.

Finally, we note the important effects of dye dipole orientation in such nanogaps. The optical field perpendicular to the metal is more than 10-fold stronger than the in-plane fields at the position of the dye. This means that in-plane dipole orientations would not emit light, while perpendicular orientations are needed for strong light-matter coupling. Here, the Cy3,5 dyes are tethered into the structure at both oligo ends, but the IRD700 is only bound from one end (Figure 1); however, no clear difference in signatures is seen. For instance, their dynamics are similar, without any signature of rapid reorientations that might be expected for the IRD700 as it diffuses around in the water pocket inside the DNAo. We thus cannot conclude anything here about the orientation, although it remains an interesting question for future work.

We also note that previous (relatively) simple theoretical models for strong coupling may not be applicable here. The very large electron density in the Au facets is situated less than the dipole size away and thus may lead to much more profound changes in the emission spectrum. The dipole–dipole interactions from electron wave packets in the emitting dye molecule and screening electrons in the metal as well as the high-density ions solvated in the water pocket will likely influence all processes. This includes Rayleigh and Raman scattering, nonradiative absorption, energy relaxation within the molecule, and phonon interactions, as well as the fundamental emission process, which operates in optical field gradients that can now be on the scale of the emitting dipole. We thus emphasize the need for further developments using these precision nanoassembly techniques.

## Conclusions

In conclusion, we show that DNA origami architectures change the electromagnetic interactions of dye molecules and Au facets, as well as the light-matter coupling. The presence of water solvating the dye leads to different optically induced forces, which we suggest arise from the local screening of the optical dipole. We show a design of well-plate scaffolded NPoM cavities, which appear to give strong coupling effects from single-molecule emitters and plasmonic modes, experimentally observed as a peak splitting in the cavity dark-field spectrum. When detuned from the plasmon energy, emitters in these plasmonic cavities couple only weakly with the cavity but give significant emission at the lower polariton energy due to hybridization. The versatility of DNA nanotechnology thus opens up a myriad of possibilities in the field of quantum technology and nanophotonics information.

## Data Availability

All data needed to evaluate the conclusions in the paper are present in the paper.
